# Absence of a dose–response relationship after intra-oropharyngeal inoculation of pigs with foot-and-mouth disease virus serotype O

**DOI:** 10.1186/s13567-026-01751-9

**Published:** 2026-04-16

**Authors:** Kira Wisnewski, Constantin Lorenz, Saskia Weber, Selma Schmidt, Ryan Waters, Phaedra Eblé, Aldo Dekker, Wilhelm Gerner, Michael Eschbaumer

**Affiliations:** 1 Institute of Diagnostic Virology, Friedrich-Loeffler-Institut, Greifswald, Germany; 2 The Pirbright Institute, Pirbright, Woking, UK; 3 Wageningen Bioveterinary Research (Part of Wageningen University & Research), Lelystad, the Netherlands

**Keywords:** Foot-and-mouth disease, FMDV, pigs, intra-oropharyngeal, inoculation, animal models, multi-laboratory study, animal challenge, pathogenesis, virus

## Abstract

Foot-and-mouth disease virus (FMDV) poses significant economic threats to livestock industries worldwide, with its serotype O being the most prevalent globally and responsible for all outbreaks in Europe in the last 25 years. This retrospective multi-centre study evaluated the dose–response relationship after intra-oropharyngeal (IOP) inoculation of pigs with FMDV O, combining independently obtained results from three research institutes. Four virus isolates were evaluated: a recombinant O/FRA/2001 virus containing the P1-coding region of O/BUL/2011, O/TAW/97, O/UKG/34/2001, and O/Manisa/TUR/69, at doses up to 8 × 10^6 TCID_50_ using IOP inoculation. In all experiments, intradermal heel bulb (IDHB) inoculation served as a positive control. Overall, results of IOP were variable and strain-dependent, without a dose–response relationship, while IDHB reliably caused infection even at low doses. No single factor responsible for the observed variation was identified. These findings indicate that, unlike IDHB, the outcome of IOP inoculation does not consistently lead to infection with FMDV.

**Supplementary Information:**

The online version contains supplementary material available at 10.1186/s13567-026-01751-9.

## Introduction

Foot-and-mouth disease (FMD) is a contagious viral disease of cloven-hoofed animals that can cause severe economic losses. The causative agent, foot-and-mouth disease virus (FMDV) is a non-enveloped single-stranded RNA virus of the species *Aphthovirus vesiculae* in the *Picornaviridae* family. The characteristic clinical manifestations of FMD, including erosions and vesiculation of the cornified epithelium within the oral cavity, on the udder and on the feet, are observed across a broad spectrum of susceptible host species, encompassing both domestic and wild ruminants as well as domestic and wild pigs. [1, 2].

There are seven antigenically distinct serotypes, which are further classified into different topotypes based on a sequence analysis of VP1, the major viral antigen [3]. Serotype O is the most prevalent and economically significant and is responsible for the majority of global outbreaks [4, 5] including the recent events in Europe. In 2025 alone, outbreaks have been reported in multiple European countries, including a small outbreak in Brandenburg, Germany, affecting 14 water buffalo. In contrast, several farms with thousands of cattle have been affected in Slovakia and Hungary. Notably, these countries had been officially free of FMD for 37 and over 50 years, respectively.

In FMD-free countries, the consequences of an FMD outbreak can be devastating with substantial economic losses due to trade restrictions and killing and disposal of affected and at-risk animals [6]. Inactivated vaccines are available, but there is no cross-protection between the serotypes and sometimes antigenic variability within serotypes limits protection within a serotype, especially when using vaccines with a low potency [7].

FMDV can be transmitted by various routes: infected animals can spread the virus through direct contact and indirect contact within the same holding. Indirect transmission can also occur through products of infected animals such as meat, milk or semen or by contaminated inanimate objects and people [6]. Infection by products of infected animals fed to pigs was most likely the cause of the huge FMD outbreak in the UK in 2001 but might also have been the route of introduction for the 2025 outbreak in water buffalo in Germany.

Since products of infected animals can spread FMDV, oral exposure to FMDV has been studied in the past [8–14]. In those studies, a wide variation in the dose required to achieve infection in 50% of exposed animals was observed, primarily depending on the virus strain, but also influenced by whether the virus was applied as a liquid suspension (resulting in a lower dose needed for infection) or mixed into solid feed. The extent to which lymphoid tissue in the oropharynx is exposed to the virus has been suggested as a key factor contributing to these differences [15]. This assumption was also the basis for Stenfeldt and colleagues to introduce intra-oropharyngeal (IOP) inoculation as an experimental method that might reflect a natural route of FMDV infection in pigs, while retaining a degree of standardization and reproducibility [12, 16].

Validation experiments with the IOP method were conducted independently at the Friedrich-Loeffler-Institut (FLI) in Germany, The Pirbright Institute in the United Kingdom, and Wageningen Bioveterinary Research (WBVR) in the Netherlands. The objective of this work is to retrospectively combine the data from these studies to evaluate the dose–response relationship of different FMDV serotype O strains following exposure targeting the oropharyngeal lymphoid tissue in pigs. This also provides an evidence-based assessment of how consistently IOP inoculation leads to infection under varying experimental conditions and across laboratories.

## Materials and methods

### Ethics statement and animals

All animal experiments were conducted under the respective national licenses and approvals at the participating institutes (Friedrich-Loeffler-Institut (FLI): LALLF M-V, file no. 7221.3-1.3-039/23, The Pirbright Institute (Home Office approved project license), and Wageningen Bioveterinary Research (WBVR): AVD401002015265). Only clinically healthy, FMDV-free pigs were used, and animals were randomly allocated to experimental groups.

At FLI, crossbred pigs (Sus scrofa domesticus), 8 weeks of age and weighing approximately 20–25 kg, were sourced from a commercial herd. At Pirbright, 12-week-old female Large White/Landrace × Hampshire pigs (30–40 kg) were used. At WBVR, 9-week-old male pigs from a multiplier herd were included. None of the pigs were vaccinated against FMDV.

Animals were housed in high-containment facilities appropriate for work with FMDV. Environmental conditions (temperature, humidity, ventilation), group size, and enrichment followed institutional standards and are described in detail in the Additional file 1: Supplementary Methods (S1).

### Virus strains and preparation

The recombinant virus O/FRA/2001-P1(O/BUL/2011) was generated using the plasmid pT7S3-O FRA, which contains the full-length cDNA of FMDV isolate O/FRA/1/2001 [17, 18]. The capsid-coding P1 region of O/FRA/1/2001 was replaced with the corresponding region of a virus originally isolated from a wild boar in Bulgaria in 2011 (isolate O/BUL/HS018-1/2011, GenBank accession number PQ619438) (Additional file 1: Supplementary Methods S2). This virus was obtained from the Bulgarian National Veterinary Service, as described by Breithaupt et al. [19]. After three passages in BHK-21 cells, the recombinant virus was used to inoculate pigs. Virus derived from the infectious clone was passaged three times in BHK-21 cells before use. Additional virus stocks were generated by first and second pig passages using vesicular lesion material collected during the acute phase of infection (Additional file 1: Supplementary Methods S3).

The porcinophilic strain O/TAW/97 as well as O/UKG/34/2001 and O/Manisa/TUR/69 were propagated according to institute-specific protocols to obtain sufficient amounts of inoculum (S3). Cell lines used for virus propagation and titration included BHK-21, LFBK-αvβ6, IB-RS-2, and primary porcine or ovine kidney cells, depending on the institute and virus strain.

All virus stocks were titrated prior to inoculation to determine the virus concentration, diluted accordingly to achieve the target doses, and re-titrated after inoculation to confirm the delivered dose. Stocks were sequenced by Sanger sequencing to exclude unintended mutations and stored at −80 °C until use. Detailed protocols for virus propagation, pig-passage preparation, titration, and sequencing are provided in the Additional file 1: Supplementary Methods (S3).

### Experimental design

The intra-oropharyngeal (IOP) route of infection was evaluated and compared to the standard intra-dermal heel bulb (IDHB) inoculation. Pigs were inoculated with different FMDV strains and doses, as summarized in Additional file 1: Supplementary Table S1. Infection outcomes were assessed clinically, virologically, and serologically.

### Inoculation procedures

Prior to inoculation, pigs were anesthetized by intramuscular injection of sedative and anaesthetic agents according to institutional protocols (Additional file 1: Supplementary methods S4). For IOP inoculation [16], pigs were placed in dorsal recumbency and 2 mL of virus suspension was deposited directly onto the tonsil of the soft palate using a blunt cannula. The inoculum was left in situ for approximately one minute to ensure sufficient contact with the oropharyngeal lymphoid tissue.

**Figure 1:**
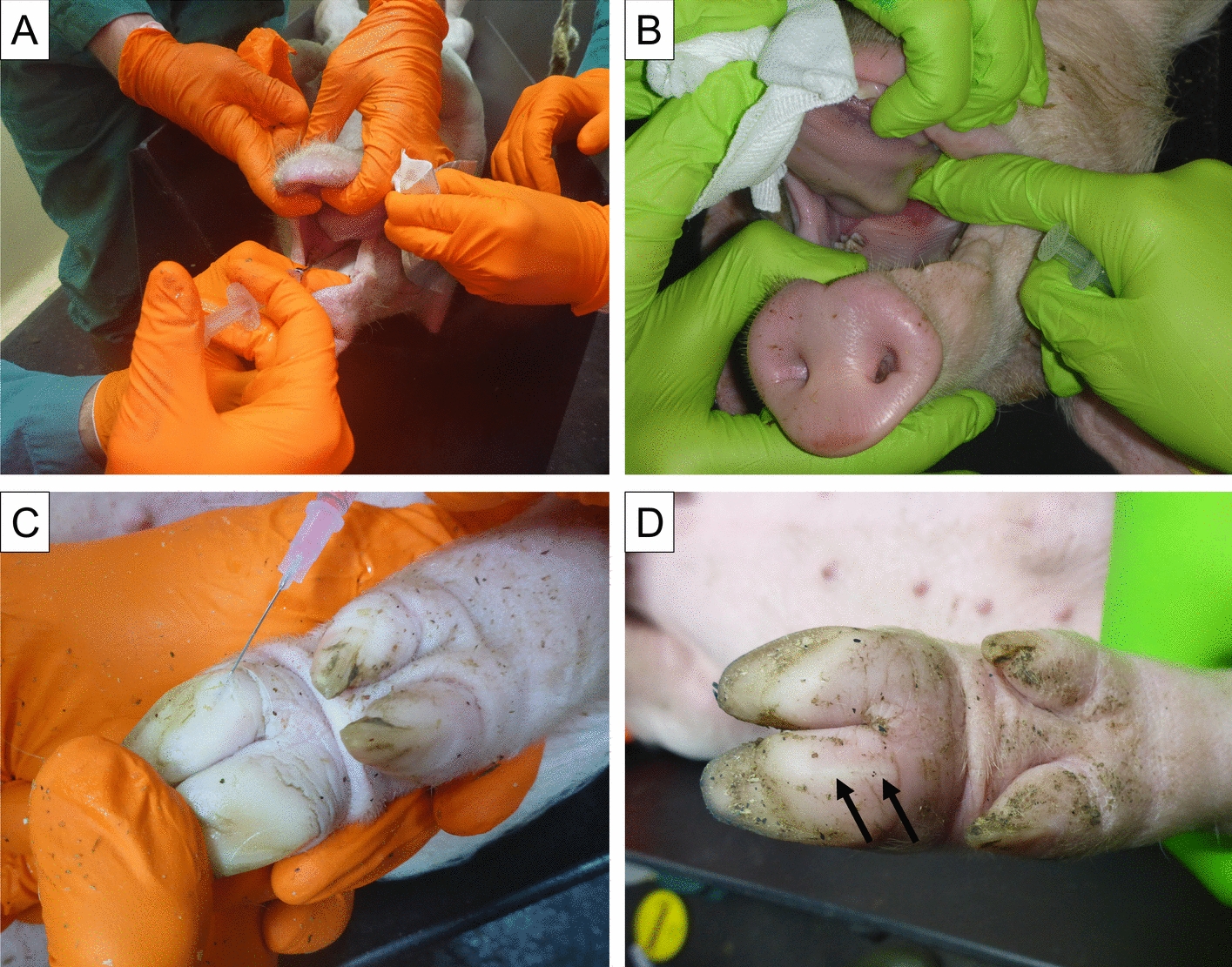
**Application of intra-oropharyngeal (IOP) and intra-dermal heel bulb (IDHB) inoculation in pigs.** **A** IOP inoculation using a blunt cannula. The inoculum is deposited in the oropharynx. **B** Close-up of the IOP inoculation, showing the placement of the inoculum onto the tonsil of the soft palate. **C** IDHB inoculation targeting the dermis of the heel bulb. The needle is inserted at a shallow angle, and the inoculum is deposited while slowly withdrawing the needle. **D** Post-inoculation image of the heel bulb, showing the injection site (black arrows) and slight tissue reaction.

For IDHB inoculation, virus suspension was injected intradermally into the heel bulb using 0.1 mL per injection site. Depending on the study, two (FLI, Pirbright) or four (WBVR) injection sites were used. IDHB inoculation served as a positive control for efficient infection (Figure 1).

### Monitoring, sampling, and clinical assessment

All pigs were monitored daily after inoculation for clinical signs of FMD, including rectal temperature, general behaviour, feed intake, lameness, and the presence of vesicular lesions on the feet, snout, or oral cavity. Blood samples and oral and/or nasal swabs were collected at predefined time points, with minor variations between institutes and trials; complete sampling schedules are provided in Additional file 1: Supplementary Table S3.

Lesion scoring was used to assess disease progression: one point was assigned for each affected digit, with three additional points for lesions in the oral cavity, on the lips, or the snout, respectively. Once an affected site was scored positive, it remained positive on all subsequent days, even if the lesions healed. In IDHB-inoculated pigs, lesions on the inoculated limb were not scored, as only viral generalization was assessed. The maximum possible scores were 19 points for IOP-inoculated pigs and 15 points for IDHB-inoculated pigs.

### Humane endpoints, euthanasia, and necropsy

Humane endpoints were applied to minimize animal suffering and included severe lameness, persistent pyrexia, marked behavioural changes, or anorexia. Scientific endpoints were defined as evidence of virus generalization, indicated by the development of FMD lesions on at least one foot other than the inoculated limb in IDHB-inoculated pigs. In the first experiment at FLI, the scientific endpoint was to monitor pigs beyond the acute phase of infection. However, if pigs became severely affected, humane endpoints were applied in accordance with the approved experimental protocols. Detailed humane endpoint criteria used at individual institutes are provided in the Additional file 1: Supplementary Methods (S5).

Euthanasia was performed under deep anaesthesia using institutionally approved protocols (Additional file 1: Supplementary Methods S5).

### Sample processing and RNA extraction

Blood samples were processed to obtain serum. Oral and nasal swabs were diluted in appropriate buffers or media. RNA was extracted from serum and swabs using magnetic-bead-based extraction systems on KingFisher platforms. An internal control RNA was included to monitor extraction efficiency and PCR inhibition. Detailed extraction volumes, buffers, and homogenization procedures are provided in the Additional file 1: Supplementary Methods (S6).

### Virus detection, isolation, and serology

Quantification of viral RNA was conducted using quantitative real-time reverse transcription PCR (RT-qPCR) targeting the 3D-coding region of the viral genome, as previously described by [20]. Ct values above 35 were considered negative. Where necessary, virus isolation was performed on susceptible cell lines to amplify virus for sequencing or titration.

Serological responses were assessed using commercially available ELISA kits targeting antibodies against FMDV non-structural proteins or serotype-specific antigens, according to the manufacturer’s instructions. The specific assays used at each institute are listed in the Additional file 1: Supplementary Methods (S6).

### Statistical analysis

Outcome and inoculation dose were analysed in a logistic regression model, using infection as a binomial result variable. Inoculation dose, viral strain and research institute were analysed as possible explanatory variables. After analysing the univariate model, a forward selection was performed with a maximum of one interaction term. The best model was chosen using Akaike’s Information Criterion [21]. The likelihood ratio test was used for determining the p value. Because observations in a group are not independent, RT-PCR and virus isolation results were not used as explanatory variables.

## Results

The intra-oropharyngeal (IOP) method was used to expose the lymphoid tissue in the pharynx of pigs to FMDV to evaluate the dose–response characteristics of different virus strains. The established method of intradermal heel bulb (IDHB) inoculation was used as a control. The studies were performed with different strains and at different locations, therefore the results of the trials are presented per research institute.

### Studies at the Friedrich-Loeffler-Institut

As different viruses and passages were used, we report the results of the separate studies. Infection was primarily assessed by lesion scores, and qPCR data served as confirmation of infection; detailed qPCR results are provided in the Additional file 2.

#### O/FRA/2001-P1(O/BUL/2011) (from infectious clone)

**Figure 2:**
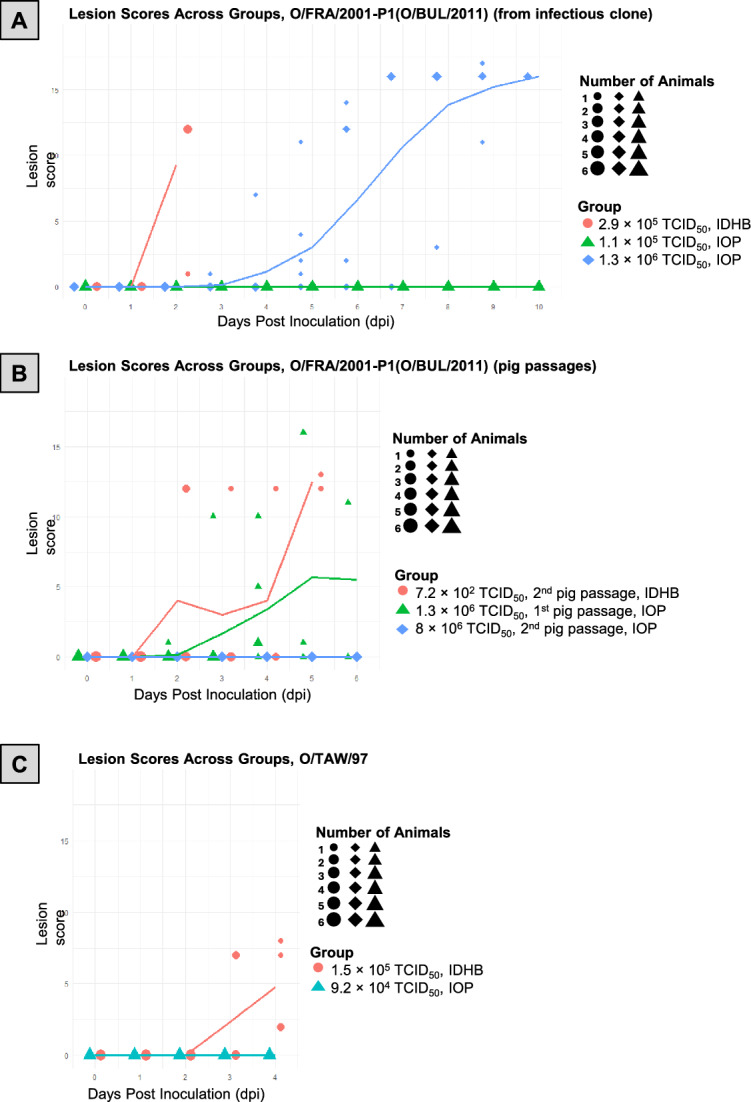
**Lesion scores per pig over time for different inoculation groups.** Lesion scores were recorded daily for each animal following the inoculation. Each data point represents the lesion score of an individual pig at a given day post-inoculation (dpi). The solid lines indicate the mean lesion score per group per day, illustrating lesion progression within each group over time. Each IOP inoculation group is shown alongside a corresponding IDHB control group for comparison. **A** O/FRA/2001-P1(O/BUL/2011) (infectious clone)—Lesion scores following IOP or IDHB inoculation with O/FRA/2001-P1(O/BUL/2011) derived from an infectious clone. **B** O/FRA/2001-P1(O/BUL/2011) (pig passages)—Lesion scores following IOP or IDHB inoculation with O/FRA/2001-P1(O/BUL/2011) after one or two passages in pigs. **C** O/TAW/97 (field isolate)—Lesion scores following IOP or IDHB inoculation with the field isolate O/TAW/97. Symbols represent individual pigs, with the symbol size indicating the number of pigs within a group that had the same lesion score on a given day. Larger symbols denote multiple pigs with identical scores on that day. Pigs that were euthanized due to FMD-related welfare concerns are no longer included in the graph after the day of euthanasia.

With the recombinant virus O/FRA/2001-P1(O/BUL/2011), the IOP method resulted in infection only at a dose of 1.3 × 10^6^ TCID_50_/2 mL. At this dose, all six pigs ultimately became infected (Figure 2A), probably caused by transmission from the first infected pig. Clinical signs appeared in one animal at 3 dpi, whereas the remaining five pigs developed disease at later time points: three at 5 dpi, one at 7 dpi, and one at 9 dpi (Table 1). Serum from the pig with immediate onset was positive in the FMDV RT-qPCR on 2 dpi and the animal developed the first detectable lesion at 3 dpi. The lowest Ct value in serum in the group was recorded at 4 dpi (Ct = 23.6) in this pig. Of the co-housed pigs, one tested RT-qPCR positive in serum at 4 dpi, another at 8 dpi (in a swab sample), and one more at 10 dpi. The remaining pigs were only RT-qPCR positive at necropsy (see Additional file 2). Due to limited sampling points (serum was only collected at 0, 2, 4 and 10 dpi), an earlier onset of viremia in some pigs cannot be excluded. No FMDV infections (no clinical signs nor positive RT-qPCR results, see Additional file 2) were seen in the groups inoculated with doses of 1.0 × 10^4^ and 1.1 × 10^5^ TCID_50_/2 mL (Table 1). The 1.3 × 10^6^ TCID_50_/2 mL group of infected pigs was initially treated with nonsteroidal anti-inflammatory drugs (meloxicam, 0.4 mg/kg) to alleviate clinical signs. Nevertheless, pigs were euthanized in accordance with welfare regulations at different time points: one pig at 8 dpi, two at 9 dpi, and the remaining pigs at 10 dpi at the planned end of the experiment.

**Table 1:** **Summary of dose and response of intra-oropharyngeal inoculation of pigs in trials**

Friedrich-Loeffler-Institut (further details in Figure 2)				
O/FRA/2001-P1(O/BUL/2011) (from infectious clone, three cell culture passages)				
Dose		Number of pigs		
	Total	FMDV RNA or virus in serum	Clinical disease	
			Immediate onset	Delayed onset
1.0 × 10^4^ TCID_50_/2 mL	6	0	0	0
1.1 × 10^5^ TCID_50_/2 mL	6	0	0	0
1.3 × 10^6^ TCID_50_/2 mL	6	6	1	5
O/FRA/2001-P1(O/BUL/2011) (1^st^ pig passage)				
1.3 × 10^6^ TCID_50_/2 mL	6	5	1	4
O/FRA/2001-P1(O/BUL/2011) (2^nd^ pig passage)				
4.6 × 10^2^ TCID_50_/2 mL	6	0	0	0
2.6 × 10^3^ TCID_50_/2 mL	6	0	0	0
1.7 × 10^4^ TCID_50_/2 mL	6	0	0	0
8.0 × 10^6^ TCID_50_/2 mL	6	0	0	0
O/TAW/97 (field isolate, two cell culture passages)				
9.2 × 10^4^ TCID_50_/2 mL	6	0	0	0
The Pirbright Institute (further details in Figure 3)				
O/UKG/34/2001				
1.0 × 10^5^ TCID_50_/2 mL	5	1	1	0
1.0 × 10^6^ TCID_50_/2 mL	5	1	0	1
Wageningen Bioveterinary Research (further details in Figure 4)				
O/Manisa/TUR/69 (pig-passaged virus)				
4.5 × 10^2^ TCID_50_/2 mL	5	1	1	0
4.5 × 10^4^ TCID_50_/2 mL	5	2	1	1

IDHB inoculation of 2.9 × 10^5^ TCID_50_/0.2 mL resulted in immediate infection of all pigs, with clinical signs observed in all pigs at 2 dpi (Table 2). Affected pigs exhibited lameness, increased body temperature, and depression. All pigs in this group were positive in the FMDV PCR at 2 dpi, with the lowest Ct value of 18.2 detected in serum (see Additional file 2). These pigs were euthanized on 2 dpi.

**Table 2:** **Summary of dose and response of intradermal heel bulb inoculation of pigs in trials**

Friedrich-Loeffler-Institut (further details in Figure 2)				
O/FRA/2001-P1(O/BUL/2011) (from infectious clone, three cell culture passages)				
Dose	Number of pigs			
		FMDV RNA or virus in serum	Clinical disease	
	Total		Immediate onset	Delayed onset
2.9 × 10^5^ TCID_50_/0.2 mL	4	4	4	0
O/FRA/2001-P1(O/BUL/2011) (2^nd^ pig passage)				
7.2 × 10^2^ TCID_50_/0.2 mL	6	6	3	3
O/TAW/97 (field isolate, two cell culture passages)				
1.5 × 10^5^ TCID_50_/0.2 mL	6	6	6	0
The Pirbright Institute (further details in Figure 3)				
O/UKG/34/2001				
1.0 × 10^5^ TCID_50_/0.2 mL	5	5	5	0
Wageningen Bioveterinary Research (further details in Figure 4)				
O/Manisa/TUR/69 (pig-passaged virus)				
0.9 × 10^4^ TCID_50_/0.4 mL	5	5	5	0

Tables 1 and 2 present infection outcomes following IOP (Table 1) and IDHB (Table 2) inoculations with various FMDV strains and doses. The applied dose was either determined by titration after infection or calculated based on the titres of the virus stock. Data include results for O/FRA/2001-P1(O/BUL/2011) (derived directly from an infectious clone or from pig passages), O/TAW/97 (field isolate), O/UKG/34/2001 (from pig passage) and O/TUR/Manisa/69 (from pig passages). The number of pigs inoculated, and the number of pigs tested positive by FMDV RT-PCR (FLI and the Pirbright Institute) or virus isolation (WBVR), as well as the time of onset of infection (immediate or delayed), is reported for each experimental condition.

#### O/FRA/2001-P1(O/BUL/2011) (1^st^ and 2^nd^ pig passages)

As the first study did not find efficient infection after IOP inoculation, we used lesion material obtained from IOP-inoculated pigs from the first study for a subsequent experiment. Here, IOP inoculation of 1.3 × 10^6^ TCID_50_/2 mL resulted in infection in five out of six pigs. One pig developed clinical signs as early as 2 dpi, while the remaining four showed delayed onset at 4 dpi (Table 1). The pig with immediate onset showed increased body temperature and lameness starting at 2 dpi and was PCR-positive in serum on the same day. Three pigs were RT-qPCR positive in serum at 4 dpi and one at 6 dpi. The pig that remained clinically unaffected was consistently PCR-negative in all serum samples. The lowest Ct value (Ct = 21.0) was recorded at 4 dpi in one of the pigs with delayed onset (see Additional file 2). Sequence analysis of the viral inoculum and viruses recovered from the first passage in pigs revealed two silent mutations in the VP2- and VP3-coding regions and one nonsynonymous mutation in VP1. Specifically, in VP1, an asparagine (N) at residue 143 was replaced by lysine (K). This substitution is located immediately upstream of the conserved RGD motif within the VP1 GH loop, with only a single amino acid separating the mutated position from the RGD sequence (original: **NVRGD**, mutated: **KVRGD**). No additional mutations were detected after the second passage in pigs.

From the study using the 1 st pig passage of O/FRA/2001-P1(O/BUL/2011), vesicular material was recovered and called the 2^nd^ pig passage of O/FRA/2001-P1(O/BUL/2011); IOP inoculation with this material using 4.6 × 10^2^, 2.6 × 10^3^, 1.7 × 10^4^, and 8.0 × 10^6^ TCID_50_/2 mL, respectively, did not result in infection (Table 1). In contrast, IDHB inoculation of 7.2 × 10^2^ TCID_50_/2 mL led to clinical infection (Table 2) as well as positive RT-qPCR results in serum of all 6 pigs. Among the IDHB-inoculated pigs, clinical signs developed early in three pigs (two at 2 dpi, one at 3 dpi), whereas the remaining three showed delayed onset at 4 dpi (*n* = 1) and 5 dpi (*n* = 2). The first two pigs developed lesions at 2 dpi (Figure 2B) and were PCR-positive in serum on the same day. The lowest Ct value was recorded in a pig with early onset of infection at 2 dpi (Ct = 22.9) (see Additional file 2).

#### O/TAW/97 (field isolate)

FMDV O/BUL/2011 is not known as a pig-adapted strain, although it was initially isolated from wild boar, for this reason we also used FMDV/O/TAW/97. IOP inoculation of 9.2 × 10^4^ TCID_50_/2 mL did not result in infection in any pig (Table 1). In contrast, IDHB inoculation of the same dose led to immediate infection in all six pigs (Table 2), all of which developed clinical signs within a similar timeframe. Lesions were observed in two pigs at 3 dpi, while the remaining four pigs developed lesions one day later (Figure 2C). Pigs 41 and 42 were PCR-positive at 3 dpi. The lowest Ct value was recorded in serum at 3 dpi (Ct = 27.8) (see Additional file 2).

### Studies at the Pirbright Institute

#### O/UKG/34/2001

**Figure 3:**
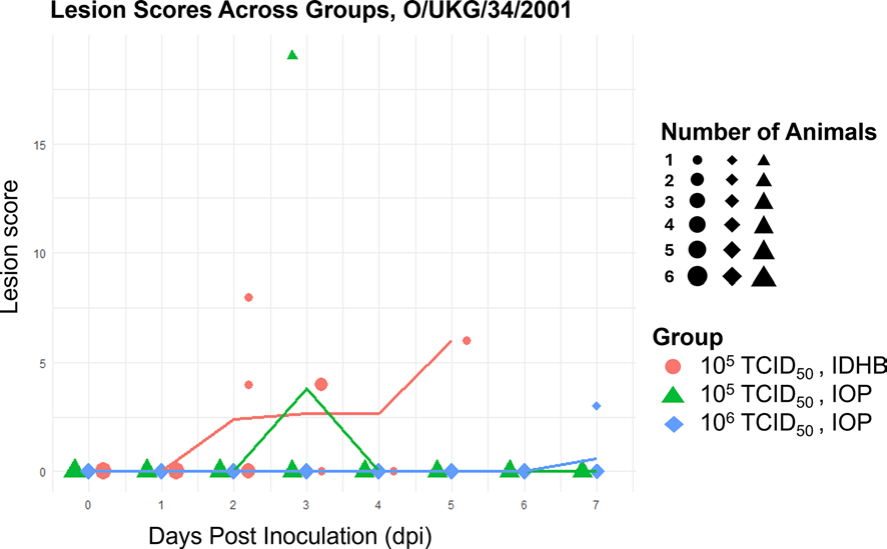
**Lesion scores per pig over time for the O/UKG/34/2001 groups.** Lesion scores were recorded daily for each pig following inoculation. Each data point represents the lesion score of an individual pig at a given day post-inoculation (dpi). The solid lines indicate the mean lesion score per group per day, illustrating lesion progression over time. Three groups were analysed: pigs inoculated via IOP with a calculated dose of 10^5^ TCID_50_, pigs inoculated via IOP with 10⁶ TCID_50_, and a corresponding IDHB control group inoculated with 10^5^ TCID_50_. Symbols represent individual pigs, with symbol size indicating the number of pigs within a group that had the same lesion score on a given day. Larger symbols denote multiple pigs with identical scores.

For the O/UKG/34/2001 virus, IOP inoculation resulted in an infection in only one out of five pigs at the calculated doses of 10^5^ and 10^6^ TCID_50_/2 mL, with clinical signs appearing immediately (10^5^ TCID_50_/2 mL) or delayed (10^6^ TCID_50_/2 mL) (Table 1). This coincided with a maximum lesion score of 18 at 3 dpi for the pig inoculated with 10^5^ TCID_50_/2 mL and a low lesion score at 7 dpi for the pig with delayed onset of infection in the 10^6^ TCID_50_/2 mL IOP group (Figure 3). These two pigs also showed FMDV 3D copy numbers of 10^8^ or higher in their sera at 4 and 7 dpi, respectively, as determined by RT-qPCR (Additional file 2). Additionally, one pig in the 10^5^ TCID_50_**/**2 mL group showed copy numbers of 10^8^ at 7 dpi but did not develop lesions. In contrast, IDHB inoculation of a calculated dose of 10^5^ TCID_50_/2 mL successfully infected all five pigs, with immediate onset of symptoms in each case (Table 2) and lesions occurring between 2 and 5 dpi (Figure 3). All pigs in this group showed 3D copy numbers of 10^8^ or higher in their sera on the day they were euthanised (Additional file 2).

### Studies at Wageningen Bioveterinary Research

#### O/Manisa/TUR/69

**Figure 4:**
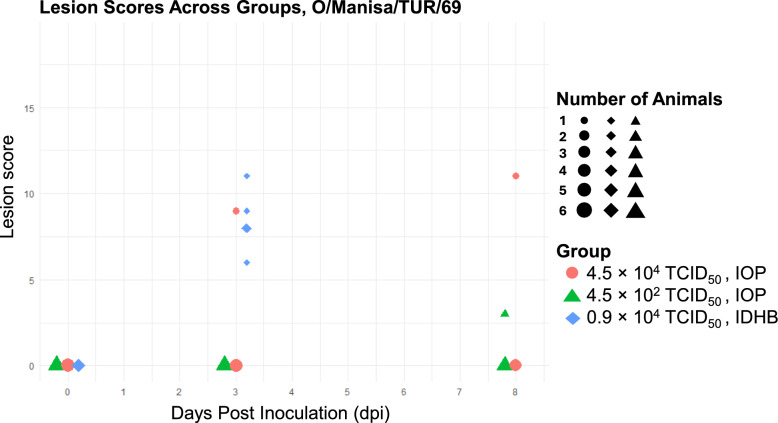
**Lesion scores per pig over time for the O/Manisa/TUR/69 groups.** Lesion scores were recorded at 0, 3 and 8 dpi for each pig following inoculation. Each data point represents the lesion score of an individual pig at a given day post-inoculation (dpi). The solid lines indicate the mean lesion score per group at the given day, illustrating lesion progression over time. Three groups were analysed: pigs inoculated via IOP at 4.5 × 10^4^ TCID_50_, pigs inoculated via IOP at 4.5 × 10^2^ TCID_50_, and a corresponding IDHB control group inoculated at 0.9 × 10 TCID_50_. Symbols represent individual pigs, with symbol size indicating the number of pigs within a group that had the same lesion score on a given day. Larger symbols denote multiple pigs with identical scores.

For the FMDV strain O/Manisa/TUR/69, lesions were only scored on day 3 and 8 after inoculation (Figure 4). IOP inoculation resulted in infection in two out of five pigs at a dose of 4.5 × 10^4^ TCID_50_/2 mL (Table 1). The first pig showed lesions on day 3 and the second one showed lesions on day 8. In this animal experiment, pigs were individually housed after inoculation. At the lower dose of 4.5 × 10^2^ TCID_50_/2 mL, only one pig had detectable lesions on day 8 (Figure 4). In contrast, IDHB inoculation with 0.9 × 10^4^ TCID_50_/0.4 mL successfully infected all five pigs (Table 2), with all pigs showing lesions by day 3.

#### Statistical analysis

We tested whether infection in a pig (immediate onset, as spread within the group was not our research objective) was correlated with viral dose, virus strain or research institute. None of these 3 variables explained infection. This means that infection was rather a random process which cannot significantly be explained by any of the explanatory variables we studied.

## Discussion

The objective of this study was to combine data from several experiments retrospectively to evaluate the dose–response relationship of different FMDV serotype O strains following exposure targeting the oropharyngeal lymphoid tissue in pigs. One would expect a dose–response relationship if infection of lymphoid tissue was the point of entry in these experiments. The tonsil of the soft palate has been suggested as a possible primary site of infection in studies reporting successful infection of pigs by direct exposure of this tissue to FMDV [15]. We used the same inoculation method as described previously [16] to assess the reproducibility of this route of infection at different research institutes with different FMDV isolates. No significant dose–response relation was found in these experiments.

The FMDV isolates used all belong to serotype O and included a recombinant O Bulgaria virus (O/FRA/2001-P1(O/BUL/2011)), O/TAW/97, which is a porcinophilic strain [22], O/UKG/34/2001, and O/Manisa/TUR/69, which was previously used in successful IOP experiments at Plum Island Animal Disease Center [16]. To confirm the infectivity of our virus isolates for pigs, they were also administered by intradermal heel bulb (IDHB) injection [23, 24], the gold standard for inoculation of pigs with FMDV. All virus isolates used in this study consistently produced clinical disease following IDHB inoculation, even at low virus doses (Table 2). Using the same virus isolates, IOP inoculation did not reliably cause infection at any of the participating research institutes (Table 1). No dose could be determined that consistently infected pigs by the oral route and statistical analysis did not find a significant dose–response relationship following IOP inoculation.

To explore whether the outcome of oral application could be improved by host adaptation of the virus, we also tested preparations of O/FRA/2001-P1(O/BUL/2011) obtained from sequential pig passages. A dose of 1.3 × 10^6^ TCID_50_/2 mL of virus, either directly from cell culture or after a single passage in pigs, resulted in infection of at least one pig. However, a higher dose of 8 × 10^6^ TCID_50_/2 mL, derived from a second pig passage, failed to cause infection. Conversely, material from the same second passage led to early infection of 50% of the pigs after IDHB inoculation, using a dose of 7.2 × 10^2^ TCID_50_/2 mL. These findings suggest that pig passages do not enhance infectivity via the IOP route, despite the assumption that prior replication in the porcine host might lead to adaptation and increase the likelihood of overcoming mucosal barriers.

Each research institute used slightly different protocols for anaesthesia, animal handling, housing, and inoculation, which may have influenced the results, but none of the institutes achieved a more consistent outcome. Moreover, no significant differences in infection outcomes were observed between them. Importantly, the absence of a clear dose–response relationship was evident across all institutes despite these methodological differences, which strengthens the conclusion that the observed variability cannot be attributed to a single experimental factor.

Pigs at FLI were anesthetized using only azaperone and ketamine, which suppressed the swallowing reflex, but was occasionally associated with increased salivation. The Pirbright Institute used a combination of azaperone, tiletamine, zolazepam, and xylazine and no swallowing occurred during the one-minute contact time. At WBVR, where the pigs were anesthetized by intramuscular injection of a mixture containing azaperone, ketamine, and xylazine, some swallowing was observed before the contact time was over. In previous studies that observed infection after IOP inoculation of pigs, the protocols were slightly different. At the Plum Island Animal Disease Center in the United States, tiletamine and zolazepam had been used in combination with ketamine and xylazine [16], while the National Institute of Animal Health in Japan [10] used xylazine in combination with pentobarbital. It is tempting to assume that the anaesthesia protocol is relevant to the outcome, but we cannot make a final conclusion since the previously used anaesthesia protocols were not followed in any of the studies reported in this paper.

The virus suspension was applied directly onto the tonsil of the soft palate and left in contact for at least one minute. At FLI, the virus suspension was diluted in tissue culture medium containing a phenol red pH indicator, enabling visual confirmation of contact with the mucosal surface of the soft palate. At Pirbright, residual inoculum was removed after the contact time, whereas at FLI and WBVR, it remained in the oral cavity and was then swallowed. Feed was offered at least one to two hours post-inoculation to avoid potential interference with virus uptake.

Another aspect that may have influenced infection outcomes is the sampling done in previous studies. For example, Stenfeldt et al. [16] sampled the oropharyngeal tonsils immediately after virus exposure using large cotton swabs. This procedure might have impacted infection success in multiple ways, e.g. by mechanically distributing the virus deeper into the crypts or by causing minor mucosal abrasions. In our experiments, low virus doses were consistently able to cause infection in pigs when inoculated into the skin by the IDHB method. This suggests that mucosal or skin damage occurring before or during the inoculation can make a huge difference.

Taken together, the duration and nature of contact between the virus and the tonsillar tissue differed slightly between the research institutes in this study and the original description of the method [16]. But since there were no significant differences between the research institutes in the current analysis, it is assumed that variations in the procedures alone cannot explain the discrepancy with previous studies.

Housing animals independently is essential in inoculation studies (as it is in potency tests). Pigs at FLI were housed in group pens, permitting horizontal transmission and complicating the differentiation between primary infection by IOP and secondary pig-to-pig infection, while pigs at the Pirbright Institute and WBVR were housed individually with visual and auditory contact only. Notably, in previous studies reporting successful infection by IOP [10, 16, 29], pigs were also kept in groups. Although group housing reflects more natural conditions, it prevents the reliable exclusion of horizontal transmission and introduces methodological and statistical limitations due to the lack of independent observations.

## Conclusions

Taken together, our findings suggest that minor procedural differences cannot account for the failure to consistently induce infection via the IOP route. The absence of a clear dose–response relationship across different institutes and experimental settings supports the robustness of this observation and argues against an effect of individual experimental parameters. Ultimately, we could not determine a dose–response relationship when using IOP inoculation, nor did we observe virus strain differences. While the method has shown promising results, our findings indicate that its robustness is more context-dependent than initially assumed.

## Supplementary Information

 **Additional file 1: Supplementary Methods.** Detailed experimental procedures, additional methodological information, and extended protocol descriptions. Supplementary Methods S1: Housing and biosecurity conditions. Supplementary Methods S2: Generation of recombinant FMD viruses. Supplementary Methods S3: Virus preparation. Supplementary Table S1: Overview of virus strains, inoculation routes, doses, and group sizes. Supplementary Methods S4: Anaesthesia protocols. Supplementary Table S2: Anaesthetic agents and dosages used for inoculation procedures. Supplementary Table S3: Overview of sampling schedules for individual experiments. Supplementary Methods S5: Humane endpoint criteria, euthanasia. Supplementary Methods S6: Laboratory methods.

**Additional file 2**: **Summary of RT-qPCR results.** Ct values and sample information for all animals and time points.

## Data Availability

No datasets were generated or analysed during the current study.

## Abbreviations

Ct: cycle threshold
DIVA: differentiating infected from vaccinated animals
dpi: days post-inoculation
FMD: foot-and-mouth disease
FMDV: foot-and-mouth disease virus
FLI: Friedrich-Loeffler-Institut
IDHB: intra-dermal heel bulb inoculation
ID_50_: median infectious dose
IOP: intra-oropharyngeal inoculation
PFU: plaque-forming units
PHID_50_: porcine heel bulb infectious dose 50%
qPCR: quantitative polymerase chain reaction
RT-qPCR: reverse transcription quantitative polymerase chain reaction
TCID_50_: 50% tissue culture infectious dose
WBVR: Wageningen Bioveterinary Research
