# Supplementary material for: Absence of a dose–response relationship after intra-oropharyngeal inoculation of pigs with foot-and-mouth disease virus serotype O
Source: Vet Res. 2026 Apr 16;57:52. doi: 10.1186/s13567-026-01751-9 (PMC13088693; doi:10.1186/s13567-026-01751-9)
Supplement: Supplementary file 1 — Additional file 1: Supplementary Methods. Detailed experimental procedures, additional methodological information, and extended protocol descriptions. Supplementary Methods S1: Housing and biosecurity conditions. Supplementary Methods S2: Generation of recombinant FMD viruses. Supplementary Methods S3: Virus preparation. Supplementary Table S1: Overview of virus strains, inoculation routes, doses, and group sizes. Supplementary Methods S4: Anaesthesia protocols. Supplementary Table S2: Anaesthetic agents and dosages used for inoculation procedures. Supplementary Table S3: Overview of sampling schedules for individual experiments. Supplementary Methods S5: Humane endpoint criteria, euthanasia. Supplementary Methods S6: Laboratory methods. [file 13567_2026_1751_MOESM1_ESM.docx]

**Supplementary Methods S1. Housing and biosecurity conditions**

Animal experiments were conducted in high-containment facilities at the Friedrich-Loeffler-Institut (FLI), The Pirbright Institute, and Wageningen Bioveterinary Research (WBVR), all of which are approved for work with foot-and-mouth disease virus (FMDV). Housing and biosecurity measures complied with the applicable national legislation and international standards for containment of FMDV.

At FLI, pigs were housed in a high-containment animal facility under veterinary biosafety level 4 conditions [25]. Animals were kept in group pens allowing social interaction. Environmental parameters, including temperature and relative humidity, were controlled according to institutional standards. Temperature (20-22 °C) and relative humidity (50-60%) are controlled. Pigs were acclimatised for one week prior to the start of the experiments. They had free access to water and were fed once a day with a commercial swine diet supplemented by hay cobs.

At The Pirbright Institute the pigs were housed in a large-animal isolation facility at The Pirbright Institute, a SAPO4 (BSL3-ag) high-containment facility compliant with UK legislation (Animals [Scientific Procedures] Act 1986) and EU Directive 2010/63/EU. Rooms were microbiologically separated with independent air supplies to prevent cross-contamination between experimental groups. Environmental conditions were maintained at 20 ± 1 °C and 55 ± 5 % relative humidity. Full decontamination of clothing using a chemical disinfectant (1:240 dilution of FAM 30; Evans Vanodine International) was applied upon exit from rooms. After challenge, additional disinfection of footwear, gloves, snares, and waterproof suits was performed when moving between pens to prevent within-room cross-contamination. Groups of five pigs were housed together until the day of challenge, after which animals were physically separated within the same room using gating and transparent barriers with sealed lower edges (Figure S1). This prevented direct contact and contact with excretions or secretions, while shared air space and visual and auditory contact were maintained to reduce stress. Pigs were housed on straw bedding, had ad libitum access to water, and were fed a weight-appropriate commercial pig grower diet twice daily.

At WBVR, pigs were housed in a large-animal containment facility meeting the “Minimum Biorisk Management Standards for FMD Laboratories” as recently revised in 2025 by the European Commission for the Control of FMD. The facility was equipped with individual air ventilation units with HEPA-filtered inlet and outlet air. Pigs were housed individually in pens separated by solid walls (1.16 m high), preventing direct physical contact while allowing visual through a window and auditory contact. Standardized feeding and watering regimes were applied.


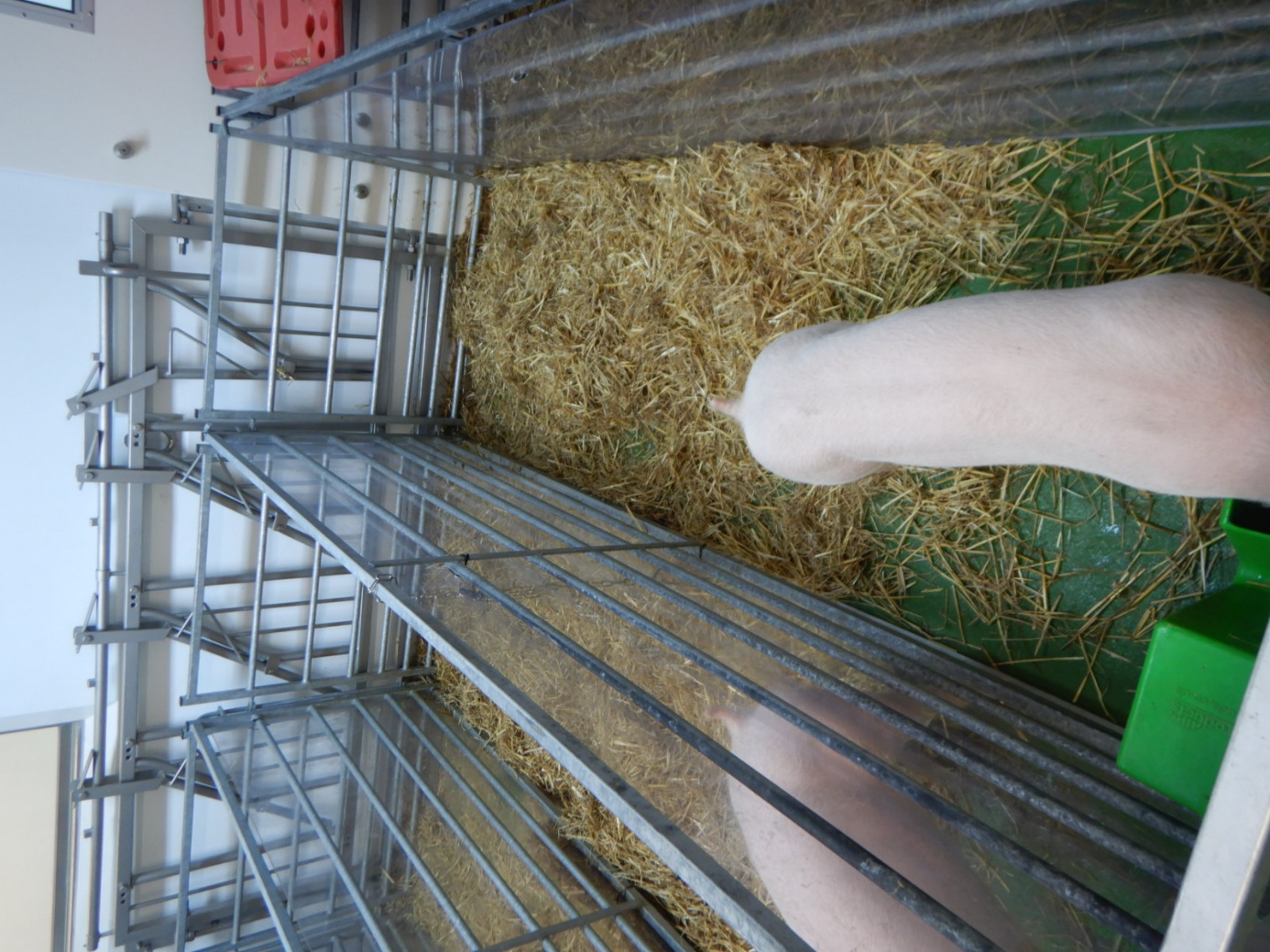


Figure S1: Housing of experimental pigs in high-containment isolation facility at The Pirbright Institute.

*Immediately post-challenge, pigs were separated from each other within the same room using transparent barriers with rubber seals along the bottom to prevent direct contact and contact with excretions or secretions from other pigs. Visual and auditory contact was maintained to reduce stress.*

**Supplementary Methods S2. Generation of recombinant FMD viruses**

For the generation of a recombinant FMD virus containing the capsid-coding P1 region of O/BUL/HS018-1/2011 in an O/FRA/1/2001 backbone, a restriction-free cloning approach [26] was used. The P1 region of the O/BUL/HS018-1/2011 virus isolate was amplified by RT-PCR after RNA extraction from cell supernatant using the QIAamp Viral RNA Kit (QIAGEN). A megaprimer of the P1 region with overhangs matching O/FRA/1/2001 was generated using AMV Reverse Transcriptase (NEB) and Phusion Hot Start Flex DNA Polymerase with the following primers: OBUL-P1-Fwd 5’ AAGCA AAGGT TCAGA AACGA CTCAG AGGTG CTGGG CAATC CAGC 3’ and OBUL-P1-Rev 5’ GACCT GACGT CAGAG AAGAA GAAAG GCCCA GGGTT GGACT CAAC 3’. In a second PCR, again using Phusion polymerase, the P1-megaprimer was combined with the pT7S3-O FRA plasmid in a 1:20 molar ratio. The PCR product was incubated with DpnI for 2 h at 37 °C to digest any leftover template DNA and then transformed into competent bacteria (NEB 10-beta Escherichia coli). BSR-T7 cells [27] in a 24-well plate were transfected with purified plasmid DNA. Each well was transfected with 500 ng of DNA using 1.5 μl of Lipofectamine 3000. For this purpose, 30 plasmids with the correct restriction fragment pattern were constructed, and after multiple rounds of transfections, three infectious clones of recombinant O/FRA/2001-P1(O/BUL/2011) were obtained. The rescued virus was subsequently passaged twice on BHK-21 cells. For each passage, 250 μl of clarified cell lysate obtained by freezing and thawing was used to infect fresh cells. CPE was observed from the second passage onwards. All virus stocks were sequenced using Sanger sequencing [28] and were stored at -80 °C. Virus titres were determined by titration on BHK-21 cells and read after 48 and 72 hours. Tissue culture infectious doses (TCID50) were calculated using the Spearman-Kaerber formula.

**Supplementary Methods S3. Virus preparation**

Pig-passaged virus material of the recombinant FMDV O/FRA/2001-P1(O/BUL/2011) was generated to evaluate the effect of in vivo passage on infectivity. Lesion material was collected from clinically affected pigs during the acute phase of infection to ensure high virus titres.

The first pig passage (O/FRA/2001-P1(O/BUL/2011), 1st pig passage) was obtained from pigs inoculated via the intra-oropharyngeal (IOP) route with 1.3 × 10^6 TCID₅₀/2 ml of virus derived from the infectious clone. Vesicular lesion material was collected during necropsy at 9 days post inoculation (dpi). Tissue was macerated using sterile sand in 20 ml serum-free Dulbecco’s Minimal Essential Medium with a mortar and pestle, followed by centrifugation at 4 °C and 2100 × g for 15 minutes.

The second pig passage (O/FRA/2001-P1(O/BUL/2011), 2nd pig passage) was generated analogously from pigs inoculated via the IOP route with 1.3 × 10^6 TCID₅₀/2 ml of the first pig passage preparation. Lesion material was collected from a pig at 4 dpi and processed using the same protocol.

For comparison, O/TAW/97, a field isolate of a porcinophilic strain, which was associated with large outbreaks on pig farms and no apparent infection in cattle, was used [29, 22]. It was propagated once in porcine IB-RS-2 cells [30] and subsequently in porcine LFBK α_V_β_6_ cells [31] for two passages to obtain sufficient amounts for pig inoculation.

The O/UKG/34/2001 virus preparation was used for inoculation of all pigs at The Pirbright Institute was generated by homogenising the epithelial component of FMD foot lesions collected at necropsy from two pigs (VO56 and 57) inoculated with an original O/UKG/34/2001 field isolate at The Pirbright Institute in 2007. An aliquot of this virus batch was titrated on BVDV-free LFBK αvβ6 cells [32] and found to have a titre of 1.6 × 10^8^ TCID_50_/ml. Virus stock was diluted in pH-verified (7.4) M25 buffer (a solution [in water] of 34.3 mmol/l Na_2_HPO_4_ and 5.7 mmol/l KH_2_PO_4_) to achieve the relevant concentrations of inoculum.

All virus stocks were sequenced using Sanger sequencing [28] and were stored at ‑80 °C. Virus titres were determined by titration on BHK-21 cells at FLI or LFBK-αvβ6 cells at The Pirbright Institute, with cytopathic effect read after 48 and 72 hours, and stocks were re-titrated after inoculation to confirm the delivered dose. Tissue culture infectious doses (TCID_50_) were calculated using the Spearman-Kaerber formula.

The virus used for inoculation at WBVR was a suspension of lesion material harvested from pigs infected by intradermal injection in the bulb of the heel with O/Manisa/TUR/69. Titrations of the virus were done on primary porcine kidney cells [33]. For conversion from PFU/ml to TCID_50_/ml, the titre in PFU/ml was divided by log_e_(2) = 0.69 (based on the Poisson distribution) [34]which is equal to adding 0.16 to the log_10_ titre (PFU/ml). The virus was titrated before the start of the experiment, but also during the study. The actual dose was based on the titre of the virus used in the study.

**Supplementary Table S1 Overview of virus strains, inoculation routes, doses, and group sizes**

| **Institute** | **Virus strain** | **Virus preparation** | **Inoculation route** | **Dose per pig** | **n** |
| --- | --- | --- | --- | --- | --- |
| FLI | O/FRA/2001-P1(O/BUL/2011) | Infectious clone (3× BHK-21) | IOP | 1.0 × 10^4^ TCID_50_ / 2 ml | 6 |
| FLI | O/FRA/2001-P1(O/BUL/2011) | Infectious clone (3× BHK-21) | IOP | 1.1 × 10^5^ TCID_50_ / 2 ml | 6 |
| FLI | O/FRA/2001-P1(O/BUL/2011) | Infectious clone (3× BHK-21) | IOP | 1.3 × 10^3^ TCID_50_ / 2 ml | 6 |
| FLI | O/FRA/2001-P1(O/BUL/2011) | Infectious clone (3× BHK-21) | IDHB | 2.9 × 10^5^ TCID_50_ / 0.2 ml | 4 |
| FLI | O/FRA/2001-P1(O/BUL/2011) | 1st pig passage | IOP | 1.3 × 10^6^ TCID_50_ / 2 ml | 6 |
| FLI | O/FRA/2001-P1(O/BUL/2011) | 2nd pig passage | IOP | 4.6 × 10^2^ TCID_50_ / 2 ml | 6 |
| FLI | O/FRA/2001-P1(O/BUL/2011) | 2nd pig passage | IOP | 2.6 × 10^3^ TCID_50_ / 2 ml | 6 |
| FLI | O/FRA/2001-P1(O/BUL/2011) | 2nd pig passage | IOP | 1.7 × 10^4^ TCID_50_ / 2 ml | 6 |
| FLI | O/FRA/2001-P1(O/BUL/2011) | 2nd pig passage | IOP | 8.0 × 10^6^ TCID_50_ / 2 ml | 6 |
| FLI | O/FRA/2001-P1(O/BUL/2011) | 2nd pig passage | IDHB | 7.2 × 10^2^ TCID_50_ / 0.2 ml | 4 |
| FLI | O/TAW/97 | Cell culture (2nd passage) | IOP | 9.2 × 10^4^ TCID_50_ / 2 ml | 6 |
| FLI | O/TAW/97 | Cell culture (2nd passage) | IDHB | 1.5 × 10^5^ TCID_50_ / 0.2 ml | 6 |
| Pirbright | O/UKG/34/2001 | Lesion material | IOP | 1.0 × 10^5^ TCID_50_ / 2 ml | 5 |
| Pirbright | O/UKG/34/2001 | Lesion material | IOP | 1.0 × 10^6^ TCID_50_ / 2 ml | 5 |
| Pirbright | O/UKG/34/2001 | Lesion material | IDHB | 1.0 × 10^5^ TCID_50_ / 0.2 ml | 5 |
| WBVR | O/Manisa/TUR/69 | Lesion material | IOP | 4.5 × 10^2^ TCID_50_ / 2 ml | 5 |
| WBVR | O/Manisa/TUR/69 | Lesion material | IOP | 4.5 × 10^4^ TCID_50_ / 2 ml | 5 |
| WBVR | O/Manisa/TUR/69 | Lesion material | IDHB | 0.9 × 10^4^ TCID_50_ / 0.4 ml | 5 |

**Supplementary Methods S4. Anaesthesia protocols**

Anaesthesia was applied prior to inoculation. All anaesthetic agents were administered intramuscularly. Each institute followed its own standard procedures, resulting in slight variations in the choice of drugs and dosages (Table S1). Pigs were fully immobilized during all inoculation procedures. At FLI and The Pirbright Institute, lighter sedation was used for intra‑oropharyngeal (IOP) inoculations, whereas a deeper anaesthesia was applied for intra‑dermal heel bulb (IDHB) inoculations to prevent any reflex movement during the precise injection. At WBVR, a single anaesthetic regimen was applied for both inoculation routes and evaluation of clinical infection 3 days after inoculation.

**Supplementary Table S2. Anaesthetic agents and dosages used for inoculation procedures**

| **Institute** | **Procedure** | **Drug** | **Dosage** |
| --- | --- | --- | --- |
| FLI | IOP | Azaperone | 2 mg/kg |
|  |  | Ketamine | 25 mg/kg |
| FLI | IDHB | Tiletamine–Zolazepam | 3 mg/kg |
|  |  | Xylazine | 4 mg/kg |
| Pirbright | IOP | Azaperone | 2 mg/kg |
|  |  | Tiletamine–Zolazepam | 0.625 mg/kg |
|  |  | Xylazine | 1 mg/kg |
| Pirbright | IDHB | Azaperone | 4 mg/kg |
|  |  | Tiletamine–Zolazepam | 2.5 mg/kg |
|  |  | Xylazine | 2 mg/kg |
| WBVR | IOP/ IDHB/ clinical evaluation | Azaperone | 2 mg/kg |
|  |  | Ketamine | 10 mg/kg |
|  |  | Xylazine | 1 mg/kg |

**Supplementary Table S3. Overview of sampling schedules for individual experiments**

| **Institute** | **Sample type** | **Sampling days post inoculation (dpi)** |
| --- | --- | --- |
| FLI | Serum, EDTA Blood | -2, 2, 4, (6)*, 14, 21 |
|  | (Nasal)**, oral swabs | -2, 2, 4, 6, 8, 10, 14, 21 |
| Pirbright | Blood, nasal, oral swabs | -1, 1, 2, 3, 4, 5, 6, 7 |
| WBVR | Blood, oral swabs | 0, 1, 2, 3, 4, 5, 6, 7 |
| All | Necropsy tissue | End point |

* After the first trial with 18 pigs inoculated via the IOP route with O/FRA/2001-P1(O/BUL/2011) (from the infectious clone), an additional blood sample was taken in the other trials on day 6 post inoculation to improve the temporal resolution of viremia detection.

**During the O/TAW/97 trial, only oral swabs were collected from the pharyngeal area due to animal welfare considerations, as the nasal sampling procedure caused noticeable distress in these pigs.

**Supplementary Methods S5. Humane endpoint criteria, Euthanasia**

Humane endpoints were applied across all studies to minimize animal suffering and were defined in accordance with institutional animal welfare policies and regulatory requirements. General humane endpoints included severe lameness, persistent pyrexia, marked behavioural changes, or prolonged anorexia.

In addition, institute-specific criteria were applied. Humane endpoints included a rectal temperature exceeding 40.5 °C for several consecutive days, inability to rise or evidence of hoof separation, delayed response to external stimuli, and refusal of food for multiple consecutive days. Humane euthanasia was also performed if multiple clinical signs occurred concurrently, even if individual criteria were not met for their full duration.

Animals reaching humane endpoints were euthanized immediately under deep anaesthesia using institutionally approved protocols. During necropsy, tissue samples were collected from vesicular lesions and at FLI also from lymph nodes, and oropharyngeal lymphoid tissues, including the tonsil of the soft palate and paraepiglottal tonsil.

At FLI all pigs were euthanised under deep anaesthesia induced by intramuscular injection of 1.5 mg/kg tiletamine, 1.5 mg/kg zolazepam, 4 mg/kg xylazine and 8 mg/kg ketamine, followed by an intracardiac injection of 24 mg/kg T61 (embutramide [200 mg/ml], mebezonium iodide [50 mg/ml], tetracaine hydrochloride [5 mg/ml]; MSD Tiergesundheit).

At The Pirbright Institute and WBVR pigs were euthanized with an overdose of pentobarbital.

**Supplementary Methods S6. Laboratory methods**

Blood samples were processed to obtain serum aliquots, and nasal and oral swabs were suspended in buffer prior to RNA extraction. At FLI, tissue samples collected at necropsy were homogenized in MEM containing antibiotics using a TissueLyser II. At The Pirbright Institute, only serum and swab samples were analysed.

RNA was extracted using magnetic bead-based kits on KingFisher robotic platforms: the NucleoMag Vet kit (Macherey-Nagel) at FLI, and the MagMAX CORE Nucleic Acid Purification Kit (Thermo Fisher Scientific) at Pirbright. An internal control RNA (IC2; [35]) was included at FLI to monitor extraction efficiency and PCR inhibition; Pirbright did not use an internal control. Extracted nucleic acid (5–10 µl) was analysed by quantitative real-time reverse transcription PCR (RT-qPCR) targeting the 3D-coding region of the FMDV genome [20]. At FLI, Ct values >35 were considered negative. At Pirbright, virus load was determined using a standard curve generated from serially diluted RNA transcripts, with Ct values proportional to FMDV RNA quantity. When needed, FLI performed virus propagation on LFBK-αvβ6 cells to amplify viral RNA for sequencing.

Serological analysis differed between institutes. At FLI, antibodies against non-structural proteins (NSP) were detected using the ID Screen FMD NSP Competition ELISA (Innovative Diagnostics), whereas Pirbright measured antibodies against structural proteins using the FMDV PrioCHECK Type O ELISA (Thermo Fisher Scientific). All assays were performed according to the manufacturers’ instructions.

At WBVR mouth swabs were vortexed in 1.5 ml of Dulbecco’s Minimal Essential Medium supplemented with 5 % foetal bovine serum and antibiotics. The swabs were kept for 15 minutes at room temperature and then centrifuged (10 minutes at 1400 × g). Virus was titrated on primary ovine kidney cells, and titres were expressed as log_10_ PFU/ml [33].
